# Overtime work, job autonomy, and employees’ subjective well-being: Evidence from China

**DOI:** 10.3389/fpubh.2023.1077177

**Published:** 2023-04-17

**Authors:** Shusheng Yang, Lijuan Chen, Xianjin Bi

**Affiliations:** ^1^School of Humanities and Foreign Languages, Qingdao University of Technology, Qingdao, Shandong, China; ^2^High-Quality Development Evaluation Institute, Nanjing University of Posts and Telecommunications, Nanjing, China; ^3^College of Public Administration, Shandong Agricultural University, Taian, Shandong, China

**Keywords:** overtime work, job autonomy, subjective well-being, involuntary overtime, China

## Abstract

**Introduction:**

Chinese workers suffer more from overtime than in many countries. Excessive working hours can crowd out personal time and cause work-family imbalance, affecting workers’ subjective well-being. Meanwhile, self-determination theory suggests that higher job autonomy may improve the subjective well-being of employees.

**Methods:**

Data came from the 2018 China Labor-force Dynamics Survey (CLDS 2018). The analysis sample consisted of 4,007 respondents. Their mean age was 40.71 (SD = 11.68), and 52.8% were males. This study adopted four measures of subjective well-being: happiness, life satisfaction, health status, and depression. Confirmation factor analysis was employed to extract the job autonomy factor. Multiple linear regression methods were applied to examine the relationship between overtime, job autonomy, and subjective well-being.

**Results:**

Overtime hours showed weak association with lower happiness (*β* = −0.002, *p* < 0.01), life satisfaction (*β* = −0.002, *p* < 0.01), and health status (*β* = −0.002, *p* < 0.001). Job autonomy was positively related to happiness (*β* = 0.093, *p* < 0.01), life satisfaction (*β* = 0.083, *p* < 0.01). There was a significant negative correlation between involuntary overtime and subjective well-being. Involuntary overtime might decrease the level of happiness (*β* = −0.187, *p* < 0.001), life satisfaction (*β* = −0.221, *p* < 0.001), and health status (*β* = −0.129, *p* < 0.05) and increase the depressive symptoms (*β* = 1.157, *p* < 0.05).

**Conclusion:**

While overtime had a minimal negative effect on individual subjective well-being, involuntary overtime significantly enlarged it. Improving individual’s job autonomy is beneficial for individual subjective well-being.

## Introduction

Economic development does not necessarily lead to reduced working hours and extended leisure time. On the contrary, working overtime has gradually become a new normal in China in recent years, especially in some industries. In 2016, a famous Chinese Internet company claimed that it was performing the “996” working-time system, which requires the employees to work from 9 a.m. to 9 p.m. for 6 days a week. This working-time system has become an implied routine among technology companies, startups, and other private businesses. This phenomenon has brought “working overtime” back into public view and aroused widespread concern, although it had existed for a long time in labor-intensive industrialization (1).

Since 1995, China has implemented a working-time system of 8 h a day, 5 days a week. Based on the standard working hours, overtime work was usually defined as working more than 40 h a week (2). A recent meta-analysis of working overtime among Chinese employees showed that work hours exhibited a fluctuating upward trend (3). Working overtime had become an unofficial part of life. Cooke surveyed small commercial and retail businesses in China and found that 22% of participants worked over 70 h a week (4). According to the China Labor Statistics Yearbook (NBS, 2021), the average weekly working hours of the 2020 Chinese urban employees were 47 h (48.1 h for males and 45.6 h for females). Among the 19 industries announced, 18 had average weekly working hours of more than 40 h, and 14 had more than 44 h. Workers in the lodging and catering industry and wholesale and retail industry worked 52.6 and 50.1 h a week, respectively, experiencing longer working hours than their counterparts in other industries. Chinese workers suffer from more severe overtime work than in other OECD countries (5).

**Figure 1:**
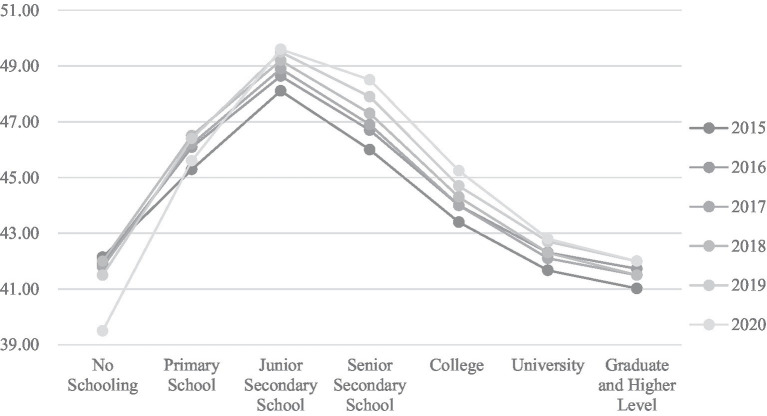
Weekly working hours of urban employed persons by educational attainment (hours/per week).

Figure 1 showed the weekly working hours per week of urban employed persons by educational attainment (NBS, 2021). This indicated that the average working hours of Chinese employees had been on the rise in recent years. While employees of all educational levels worked overtime in general, there was a gap between employees of different education levels. The average weekly working hours of no-schooling and primary education employees were lower than those of higher education employees, mainly because most of them worked in agriculture, forestry, animal husbandry, and fishing industries, where the average working hours were lower. Employees with junior high and high school education levels had the highest average weekly work hours. They are low-educated and low-skilled, work mainly in labor-intensive industries and have to face severe overtime.

Tsai et al. examined the convergence and divergence pattern of working overtime in four East Asia countries/districts: Japan, South Korea, Taiwan, and China (6). They found that professional employees experienced more working overtime in Japan, while workers in disadvantaged situations (e.g., migrant workers) experienced long work hours in China. Compared to their full-time counterparts, casual workers appeared to work longer to compensate for their wages. Based on Self-determination theory, work motivation can be classified into internal motivation (e.g., participating in activities, gaining more knowledge, improving skills, and building social connections) and external motivation (e.g., earning rewards and avoiding punishment) (7–9). Therefore, Liu et al. proposed an overtime motivation model consisting of internal and external motivation factors (5). Multiple factors can drive overtime at the same time. Working overtime is sometimes not optional for some employees, even though others choose or even pursue overtime for some reason. Previous studies documented that considerable working overtime is mandatory and often imposed by their supervisors without advance notice (10, 11).

Subjective well-being (SWB) includes cognitive assessment of whole life satisfaction and some particular aspects of life, such as job satisfaction, health status, and positive and negative emotional responses to ongoing life (12, 13). In recent years, overtime has been considered a key factor influencing people’s well-being. Previous empirical literature suggested a negative relationship between overtime and well-being (14). Studies indicated that working overtime was related to increased work-life conflict (15, 16), job burnout (17), fatigue, depression, and stress (18). As a result, working overtime impairs workers’ well-being (19).

Long working hours were associated with psychological impairments in various circumstances. Ahn noted that less work hours induced individuals to exercise regularly and decreased the likelihood of smoking (20). There were correlations between overwork and smoking, alcoholism, and overeating (21, 22). A meta-analysis reported a negative association between weekly work hours and health (23). Van der Hulst (24) found that long work hours might trigger physical diseases, such as cardiovascular and diabetes, and increase self-reported subjective fatigue and declining health. Caruso et al. concluded that working overtime was related to ill health, higher injury rates, and higher mortality (25). The adverse effects of long working hours on the physical health of workers also included shortened sleep, musculoskeletal disorders, psychosomatic symptoms, cerebrovascular diseases, hypertension, obesity, risk of stroke, and other diseases (26–31).

The negative association between long work hours and psychological consequences is well documented. Excessive overtime work was related to a higher risk of mental health problems (32). Virtanen et al. conducted a meta-analysis and concluded that long working hours were positively associated with depressive symptoms, and this association was stronger in Asian countries than in others (33). Ma’s study indicated that long working hours had positive and significant relationship with the risk of mental illness, and the effect was more significant for women, white-collar workers, and employees in micro-firms, compared with their counterparts (34). Sex-stratified analysis showed that working long hours increased the odds of depression and anxiety symptoms among females (35). Besides depression and anxiety symptoms, other psychological issues, such as stress and suicidal tendencies, were also proven to be related to long working hours (36, 37).

Though overtime had an adverse effect on well-being, the effect might be offset to some extent when workers volunteered to work overtime. A study highlighted the risks for employees’ well-being associated with unregulated extended work availability – especially when it is perceived as illegitimate (38). Employees who work long hours against their will are more likely to experience a lower level of subjective well-being than those who choose to work overtime. Karhula et al. suggested that tight deadlines, performance pressure, weekend work and lack of working time autonomy are linked to impaired well-being among health care employees (39).

Self-determination theory assumes that people have basic psychological needs for autonomy, relatedness, and competence, which are essential for well-being (40). Research has suggested a relationship between autonomy and subjective well-being (41). Job autonomy, often measured by the extent to which individuals can decide what to do and how and when to do it (42), can increase workers’ sense of control (43) and decrease work-life conflict (16), thereby serving as a significant predictor of well-being. Yu and Leka highlighted that control over time-off was related to decreased depression, anxiety, stress and work–family conflict, while control over daily hours was related to decreased stress and work–family conflict (41). Previous literature showed that autonomy was one of the strongest predictors of individuals’ life satisfaction and happiness. It exerted direct and indirect impacts on different SWB facets in Lebanese nurses, including life satisfaction, happiness, and positive and negative emotions (44). Bastida, Neira, and Lacalle-Calderon analyzed data from the European Social Survey and found that job discretion influenced SWB, which differed between males and females (45).

The relationship between overtime, job autonomy, and subjective well-being has been well established with the growing literature. However, the evidence of this relationship was mostly limited to western countries and East Asia countries such as Japan and Korea. Few studies have been conducted on overtime, job autonomy, and well-being in China, where workers are experiencing serious overtime and less job autonomy. Moreover, in China, though overtime is a common problem faced by employees in almost all industries in China, it is more serious for low-educated and low-skilled employees, which is considerably different from the high-educated study populations (e.g., medical staff and teachers) in previous studies in other countries. In these respects, this study aims to fill the research gap by addressing the association between overtime-related factors (particularly overtime hours, voluntary overtime, and job autonomy) and subjective well-being among Chinese employees. In view of this, the following hypotheses were formulated:

*H1*: Overtime hours have a negative relationship with the subjective well-being of employees.

*H2*: Job autonomy has a positive relationship with the subjective well-being of employees.

*H3*: There are interactive effects of overtime hours and job autonomy on the subjective well-being of employees who work overtime.

## Materials and methods

### Sample

We obtained data from the 2018 China Labor-force Dynamics Survey (CLDS 2018), a comprehensive survey focusing on the labor force aged 15–64 in China’s urban and rural areas. CLDS uses a multi-stage, multi-level probability sampling method proportional to the size of the labor force, which covers 28 provinces and cities in China (excluding Xinjiang, Tibet, and Hainan). It collects information on respondents’ education, employment, labor rights, occupational mobility, occupational protection and health, and occupations. The survey’s core information focuses on the current work situation and changes in satisfaction and happiness. Moreover, the survey collects community information about the labor force and family information such as demographic structure and financial and property status. CLDS 2018 contains a sample size of 381 communities/villages, 9,868 households, and 16,537 individuals. The present study retained 4,081 respondents from the survey who provided information on all the key variables: overtime hours, job autonomy, voluntary overtime, and subjective well-being. After excluding samples with missing values in other variables, our analysis sample was 4,007. Their mean age was 40.71 (SD = 11.68), and 52.8% were males. Given that the voluntary overtime variable was not applicable to the non-overtime group, it was only for the overtime group, with a subsample size of 1,188 (Mean = 0.31, SD = 11.68).

### Survey questions and variables

*Subjective well-being*: Literature has suggested two common measurements of subjective well-being. One is life satisfaction orientation, including the assessment of happiness and life satisfaction in general and in specific aspects (46, 47). The other is health psychology orientation, which contains positive and negative emotions related to psychological well-being (48, 49). Therefore, the present study employed four indicators of subjective well-being: happiness, life satisfaction, health status, and depression. In the CLDS survey, happiness was measured using the question “In general, do you think your life is happy?” with a 5-point Likert scale answer (from 0 = very unhappy to 4 = very happy). Life satisfaction was obtained using the question, “In general, are you satisfied with your life?” Options were scored from 0 (very unsatisfied) to 4 (very satisfied). Health status was constructed based on self-reported health, ranging from 0 to 4, with higher scores representing better health status. Depression was measured using the Center for Epidemiologic Studies Depression Scale (CES-D), which was developed by Radloff in 1977 and verified as a valid and reliable instrument for measuring depression among Chinese individuals (50–52). CES-D has 20 measurement items. Options were coded as “0” for basically no, “1” for rarely, “2” for often, and “3” for almost often. Scores were summed at 0–60; the higher the score, the greater the depression symptoms.

*Overtime hours* referred to the total overtime hours worked by the respondents in the previous month. To reduce sample attrition, we assigned 0 to the overtime hours of workers who did not work overtime in the previous month.

*Voluntary overtime* referred to whether the respondents have the option of working overtime.

*Job autonomy* was measured using the following three questions: “To what extent is the work content determined by yourself?,” “To what extent is the progress of the work determined by yourself?,” and “To what extent is the workload/intensity determined by yourself?” Responses ranged from 1 (totally up to others) to 3 (totally up to oneself). Principal component factor analysis was employed to extract a common factor from these three questions (KMO = 0.776, Cronbach’s α = 0.953). Table 1 shows the rotated standardized factor loadings of the observable variables.

**Table 1:** Standardized factor loadings of the observable variables.

Latent construct	Observed variables	Factor loading
Job autonomy	To what extent is the work content determined by yourself?	0.910
	To what extent is the progress of the work determined by yourself?	0.929
	To what extent is the workload/intensity determined by yourself?	0.929

Our regression models also considered covariates, mainly socioeconomic and demographic indicators. The control variables included gender (1 = male, 0 = female), age (continuous, ranging from 15 to 74), marital status (1 = unmarried, 0 = married), membership of the Communist Party of China (CPC) (1 = yes, 0 = no), hukou status (1 = urban, 0 = rural), educational years (continuous, ranging from 0 to 19), and income (personal wage income in 2017, continuous and logarithmic, ranging from 0 to 12.35). Income was winsorized at the 1 and 99% levels to reduce the impact of outliers.

### Statistical analysis

This study extracted the job autonomy factor using confirmatory factor analysis (CFA) based on related questions. The bivariate correlation method was performed to analyze the associations between the indicators of subjective well-being and other factors that might impact them. Further analyses were conducted to test the relationships between overtime hours, job autonomy, and subjective well-being using multiple linear regression approaches. Finally, the study examined the associations between overtime hours, voluntary overtime and subjective well-being among overtime workers. The latter two analyzes only involved the overtime group, with a sample size of 1,188. All analyses were conducted using Stata 16.0.

## Results

The means, standard deviations (SDs), and associations between variables are shown in Table 2. The means of happiness, life satisfaction, and health status were above the average (2.889, 2.806, and 2.896, respectively). The mean depression was 6.619 (SD = 8.939). The mean overtime hours were 22.248 in the previous month. Mean job autonomy was below zero, which means employees had lower job autonomy than other labor market participants such as employers, self-employed, and farmers. Most respondents were married, had rural hukou status, and had no affiliation with CPC.

**Table 2:** General characteristics of variables and bivariate correlation (*N* = 4,007).

	Mean	S.D.	Happiness	Life satisfaction	Health status	Depression	Overtime hours	Voluntary overtime	Job autonomy	Gender	Age	Marital status	Member-ship of CPC	Hukou status	Educational years	Income
Happiness	2.889	0.836	1.000													
Life satisfaction	2.806	0.859	0.764^***^	1.000												
Health status	2.896	0.837	0.233^***^	0.252^***^	1.000											
Depression	6.619	8.393	−0.248^***^	−0.263^***^	−0.254^***^	1.000										
Overtime hours	22.248	41.071	−0.063^***^	−0.083***	−0.067^***^	0.037^*^	1.000									
Voluntary overtime	0.312	0.464	−0.093^**^	−0.103^***^	−0.084^**^	0.074^*^	−0.019	1.000								
Job autonomy	−0.765	0.861	0.079^***^	0.077^***^	0.056^***^	−0.037^*^	−0.042^**^	−0.185^***^	1.000							
Gender	0.528	0.499	−0.020	−0.011	0.021	−0.083^***^	0.068^***^	−0.050^+^	−0.006	1.000						
Age	40.710	11.680	−0.025	0.026^+^	−0.162^***^	−0.014	0.039^**^	0.049^+^	−0.054^***^	0.144^***^	1.000					
Marital status	0.183	0.387	−0.073^***^	−0.077^***^	0.076^***^	0.043^**^	−0.023	−0.031	0.003	−0.025	−0.458^***^	1.000				
Membership of CPC	0.153	0.360	0.104^***^	0.104^***^	0.019	−0.043^**^	−0.093^***^	0.009	0.041^**^	0.101^***^	0.079^***^	−0.053^***^	1.000			
Hukou status	0.345	0.475	0.046^**^	0.052^***^	0.028^+^	0.004	−0.136^***^	0.136^***^	−0.004	−0.018	0.035^*^	0.019	0.173^***^	1.000		
Educational years	11.519	3.735	0.119^***^	0.123^***^	0.136***	−0.051^**^	−0.237^***^	0.049^+^	0.084^***^	0.000	−0.324^***^	0.179^***^	0.315^***^	0.366^***^	1.000	
Income	10.297	1.849	0.035^*^	0.038^**^	0.073^***^	−0.071^***^	−0.057^***^	−0.071^**^	0.035^*^	0.131^***^	0.031^+^	−0.091^***^	0.117^***^	0.120^***^	0.188^***^	1.000

^***^p < 0.001, ^**^p < 0.01, ^*^p < 0.05, ^+^*p* < 0.1; For the variable “voluntary overtime,” the sample size is 1,188, including only overtime workers.

There was a very strong correlation between happiness and life satisfaction (*r* = 0.764, *p* < 0.001). Overtime hours were negatively related to happiness (*r* = −0.063, *p* < 0.001) and life satisfaction (*r* = −0.083, *p* < 0.001). Job autonomy was positively related to happiness (*r* = 0.079, *p* < 0.001), life satisfaction (*r* = 0.077, *p* < 0.001), and health status (*r* = 0.056, *p* < 0.001); it was negatively related to depression (*r* = −0.037, *p* < 0.05). Regarding controlled variables, males showed fewer depressive symptoms than females. Older age was associated with bad health status. Being unmarried showed negative relationships with happiness and life satisfaction and positive relationships with health status and depression. Membership in CPC showed a similar pattern to educational years; both were positively related to happiness, life satisfaction, and health status and negatively related to depression. Urban hukou status was associated with higher happiness and life satisfaction. Income was positively associated with happiness, life satisfaction and health status and negatively associated with depressive symptoms.

Job autonomy and voluntary overtime were considered two facets of job control. To examine the relationship between overtime hours and job autonomy and well-being, we tested the independent and interactive effects of overtime hours and job autonomy, overtime hours and voluntary overtime, respectively. Table 3 presents the results of multiple linear regression analysis of overtime hours and job autonomy. Overtime hours showed significantly correlate with happiness, life satisfaction, and health status. The associations between overtime hours and happiness, life satisfaction and health status were modest (*β* = −0.002, *p* < 0.01; *β* = −0.002, *p* < 0.01; *β* = −0.002, *p* < 0.001, separately). Job autonomy was positively related to happiness (*β* = 0.093, *p* < 0.01), life satisfaction (*β* = 0.083, *p* < 0.01). The correlations between job autonomy and health and depression did not reach significance (see Model 1, Model 3, Model 5, and Model 7 in Table 3). There were significant interaction effects of overtime hours and job autonomy on health status. More overtime hours would offset the positive effect of job autonomy on health status (see Model 6 in Table 3). The interaction effects of overtime hours and job autonomy on happiness, life satisfaction, and depression did not reach significance (see Model 2, Model 4, and Model 8 in Table 3).

**Table 3:** Multiple linear regression analysis of overtime hours and job autonomy.

	Happiness		Life satisfaction		Health status		Depression	
	(1)	(2)	(3)	(4)	(5)	(6)	(7)	(8)
Overtime hours	−0.002^**^	−0.001	−0.002^**^	−0.002^*^	−0.002^***^	−0.003^***^	0.005	0.006
	(0.001)	(0.001)	(0.001)	(0.001)	(0.001)	(0.001)	(0.006)	(0.009)
Job autonomy	0.093^**^	0.071^+^	0.083^**^	0.080^*^	0.025	0.066^+^	−0.161	−0.202
	(0.029)	(0.037)	(0.031)	(0.039)	(0.029)	(0.036)	(0.301)	(0.381)
Overtime hours × Job autonomy		0.001		0.000		−0.001^+^		0.001
		(0.001)		(0.001)		(0.001)		(0.007)
Gender (female = 0)	−0.073	−0.075	−0.081	−0.081	0.043	0.047	−1.658^**^	−1.662^**^
	(0.052)	(0.052)	(0.054)	(0.054)	(0.050)	(0.050)	(0.529)	(0.530)
Age	−0.001	−0.001	0.004	0.004	−0.009^***^	−0.009^***^	−0.001	−0.001
	(0.003)	(0.003)	(0.003)	(0.003)	(0.003)	(0.003)	(0.028)	(0.028)
Marital status (married = 0)	−0.155^*^	−0.158^*^	−0.063	−0.063	0.077	0.083	0.615	0.608
	(0.075)	(0.075)	(0.078)	(0.078)	(0.073)	(0.073)	(0.770)	(0.771)
Membership of CPC (no = 0)	0.191^**^	0.194^**^	0.202^**^	0.202^**^	0.018	0.012	0.499	0.505
	(0.068)	(0.068)	(0.071)	(0.071)	(0.066)	(0.066)	(0.696)	(0.697)
Hukou status (rural = 0)	−0.041	−0.041	−0.066	−0.066	−0.095^+^	−0.095^+^	1.210^*^	1.210^*^
	(0.057)	(0.057)	(0.059)	(0.059)	(0.055)	(0.055)	(0.584)	(0.585)
Educational years	0.013	0.012	0.025^**^	0.025^**^	0.004	0.005	−0.055	−0.055
	(0.009)	(0.009)	(0.009)	(0.009)	(0.008)	(0.008)	(0.088)	(0.089)
Income (ln)	0.001	0.000	−0.002	−0.002	0.008	0.010	−0.195	−0.196
	(0.015)	(0.015)	(0.015)	(0.015)	(0.014)	(0.014)	(0.150)	(0.150)
Constant	2.956^***^	2.955^***^	2.504^***^	2.504^***^	3.150^***^	3.152^***^	10.068^***^	10.066^***^
	(0.225)	(0.225)	(0.233)	(0.233)	(0.218)	(0.217)	(2.297)	(2.298)
*N*	1,188	1,188	1,188	1,188	1,188	1,188	1,188	1,188
*R* ^2^	0.037	0.038	0.045	0.045	0.035	0.038	0.017	0.017

***p < 0.001, **p < 0.01, *p < 0.05, ^+^*p* < 0.1; Standard errors are in parentheses.

We analyzed the relationship between overtime hours and voluntary (or involuntary) overtime and employees’ subjective well-being. The results were presented in Table 4. The results of Model 1, Model 3, Model 5, and Model 7 in Table 4 indicated a significant negative correlation between involuntary overtime and subjective well-being. Involuntary overtime might decrease the level of happiness (*β* = −0.187, *p* < 0.001), life satisfaction (*β* = −0.221, *p* < 0.001), and health status (*β* = −0.129, *p* < 0.05) and increase the depressive symptoms substantially (*β* = 1.157, *p* < 0.05). As for the interaction effect of overtime hours and voluntary overtime, the correlation of interaction term with life satisfaction was significant at the 0.1 level (see Model 4 in Table 4). Overtime hours and involuntary overtime both reduced employees’ life satisfaction, and the longer the involuntary overtime, the deeper the reduction in individual’s life satisfaction. The interaction effects of overtime hours and voluntary overtime on happiness, health status, and depression did not reach significance (see Model 2, Model 6, and Model 8 in Table 4).

**Table 4:** Multiple linear regression of overtime hours and voluntary overtime.

	Happiness		Life satisfaction		Health status		Depression	
	(1)	(2)	(3)	(4)	(5)	(6)	(7)	(8)
Overtime hours	−0.002^**^	−0.002^*^	−0.002^**^	−0.001	−0.002^***^	−0.002^*^	0.005	−0.001
	(0.001)	(0.001)	(0.001)	(0.001)	(0.001)	(0.001)	(0.006)	(0.008)
Voluntary overtime (yes = 0)	−0.187^***^	−0.161^*^	−0.221^***^	−0.140^*^	−0.129^*^	−0.083	1.157^*^	0.585
	(0.054)	(0.068)	(0.056)	(0.070)	(0.052)	(0.066)	(0.553)	(0.695)
Overtime hours × Voluntary overtime		−0.001		−0.003^+^		−0.002		0.019
		(0.001)		(0.001)		(0.001)		(0.014)
Gender (female = 0)	−0.072	−0.072	−0.082	−0.083	0.040	0.039	−1.630^**^	−1.619^**^
	(0.052)	(0.052)	(0.054)	(0.053)	(0.050)	(0.050)	(0.528)	(0.528)
Age	−0.001	−0.001	0.005+	0.005+	−0.009^***^	−0.009^***^	−0.004	−0.003
	(0.003)	(0.003)	(0.003)	(0.003)	(0.003)	(0.003)	(0.028)	(0.028)
Marital status (married = 0)	−0.148^*^	−0.149^*^	−0.059	−0.061	0.076	0.075	0.631	0.648
	(0.075)	(0.075)	(0.078)	(0.078)	(0.073)	(0.073)	(0.768)	(0.768)
Membership of CPC (no = 0)	0.186^**^	0.189^**^	0.196^**^	0.203^**^	0.014	0.018	0.531	0.481
	(0.068)	(0.068)	(0.070)	(0.070)	(0.066)	(0.066)	(0.695)	(0.696)
Hukou status (rural = 0)	−0.026	−0.025	−0.045	−0.044	−0.081	−0.081	1.079^+^	1.073^+^
	(0.057)	(0.057)	(0.059)	(0.059)	(0.056)	(0.056)	(0.587)	(0.587)
Educational years	0.016^+^	0.016^+^	0.028^**^	0.028^**^	0.005	0.005	−0.064	−0.059
	(0.009)	(0.009)	(0.009)	(0.009)	(0.008)	(0.008)	(0.088)	(0.088)
Income (ln)	−0.002	−0.002	−0.006	−0.007	0.006	0.005	−0.172	−0.163
	(0.015)	(0.015)	(0.015)	(0.015)	(0.014)	(0.014)	(0.150)	(0.150)
Constant	2.901^***^	2.901^***^	2.476^***^	2.475^***^	3.164^***^	3.164^***^	9.856^***^	9.862***
	(0.222)	(0.222)	(0.230)	(0.229)	(0.215)	(0.215)	(2.266)	(2.266)
*N*	1,188	1,188	1,188	1,188	1,188	1,188	1,188	1,188
*R* ^2^	0.039	0.039	0.051	0.054	0.039	0.040	0.021	0.022

***p < 0.001, **p < 0.01, *p < 0.05, ^+^*p* < 0.1; Standard errors are in parentheses.

## Discussion

The research field of well-being is devoting much effort to identifying the influencing factors in the workplace. Company-oriented long working hours have become an important strategy for coping with human resource shortages and maximizing the exploitation of human resources, which are considered related to lower well-being. Although overtime patterns are heterogeneous across individuals in different industries and with different socio-demographic characteristics, they also have some features in common. Therefore, we introduced overtime and job control, including worktime control and job autonomy, into the study on employees’ subjective well-being. Our study estimated the influence of overtime hours, voluntary overtime, and job autonomy on subjective well-being among employees in China.

The associations between overtime hours and subjective well-being were modest in three aspects (happiness, life satisfaction, and self-reported health status) among Chinese workers. Though the relationship between overtime and well-being was documented in previous literature, a few studies found no evidence for these workhour effects (53, 54). Sparks et al. reported that the extent of association between overtime and adverse health outcome is modest (55). In this regard, the correlation between overtime hours and well-being remained suspicious. More work should be done across different population groups before firm conclusions can be drawn. Working overtime would squeeze time for family activities, leisure, and fatigue recovery and interfere with work-home balance, thereby lowering workers’ well-being. However, this association was relatively weak in this study, not exerting a large effect on individuals’ subjective well-being. For some employees, overtime might compensate for their low income. Given that overtime premium was relatively better in China than in other countries, overtime premium might account for 50% of employees’ salary (6, 56). The increase in income might offset the negative impact of overtime, ascribed to the positive relationship between income and well-being (57, 58).

There was a significant relationship between job autonomy and employees’ well-being. The more control employees had over their work content, work progress, and workload, the better their well-being (i.e., higher levels of happiness, life satisfaction, and health status). The results were consistent with the self-determination theory. Meeting the basic psychological needs for autonomy would increase the employees’ well-being. The association between job autonomy and subjective well-being might differ across socio-cultural contexts. Ghazzawi et al. found that the association between job autonomy and subjective well-being can only be achieved through engagement in increasing structural job resources and increasing challenging job demands, as collectivistic culture decreases employees’ perceptions of work autonomy (44).

Compared to voluntary overtime, involuntary overtime was detrimental to well-being. Involuntary overtime was related to a lower level of happiness, life satisfaction, self-reported health status, and more depression symptoms. Employees who volunteered to work long hours might make arrangements for families in advance and be mentally prepared for overtime, thus reducing the work-home conflict and offsetting the negative effect on well-being (59, 60). Employer-mandated overtime has an additional detrimental effect on individuals’ well-being (61). Employees who are self-driven and have higher achievement motivation work long hours more often (62, 63). For these people, rewards or promotion opportunities accompanied by working overtime would also counteract the negative effect on well-being. A study on work-nonwork balance also indicated that involuntary overtime had a negative effect, and voluntary overtime had a positive direct effect but a negative indirect effect (64).

### Strengths and limitations

Chinese culture is distinctive from other countries (65). Lockett proposed four main features of Chinese culture related to organization, including group orientation and respect for hierarchy (66). Thus, the overtime culture is widely accepted and has even become an important part of code in many corporates in China (67, 68). In China, overtime hours show only a moderate correlation with the well-being of employees. Meanwhile, whether voluntarily working overtime is salient for their well-being. Involuntary overtime poses more work-home imbalance to people who do not accept overtime culture, which is detrimental to their well-being. In addition, as material needs have been met, job design with high autonomy is supposed to be an important means to improve employees’ well-being. This research systematically analyzed overtime, voluntary overtime, job autonomy, and employees’ well-being, supplementing empirical evidence from China in related research fields. Given the deficient implementation of China’s labor security system, overtime and job autonomy should receive more public attention.

This study has some limitations. First, due to data limitations, we could not identify specific overtime patterns, such as work shifts, precariousness, and weekend overtime, which have been proven related to well-being in some literature. Second, the study could not assess the causal mechanisms of overtime, job autonomy, and well-being due to the limitation of the cross-sectional data. Extending the observation period and systematically testing key time thresholds for the development of overtime and job autonomy could have increased confidence in the findings. Third, overtime and job autonomy might have a lag effect or a cumulative effect on employees’ well-being when overtime was considered unacceptable and the level of job autonomy was low. However, it was difficult to construct the function between overtime, job autonomy, and well-being under the existing theoretical framework. Finally, it is important to note that although subjective well-being had some stability over time, short-term subjective well-being was susceptible to individual life events and perceptions at the moment, which might interfere with its relationship to work characteristics. In addition, the relationship between overtime, job autonomy, and well-being might be moderated by other work or individual characteristics, which can be explored in future studies.

## Conclusion

This study estimated the impact of overtime, voluntary overtime, and job autonomy on the subjective well-being of Chinese employees. In contrast to voluntary overtime, involuntary overtime is detrimental to well-being. It was associated with lower levels of happiness, life satisfaction, self-reported health status and more depressive symptoms. The results also showed a significant relationship between job autonomy and employees’ well-being. The more control employees have over the content of their work, work progress and workload, the better their well-being.

## Data availability statement

The raw data supporting the conclusions of this article will be made available by the corresponding author, without undue reservation.

## Author contributions

SY and XB: conceptualization and writing — original draft. LC: formal analysis and review and editing. All authors contributed to the article and approved the submitted version.

## Funding

This research was supported by Humanities and Social Science Fund of Ministry of Education of China (22YJA840003).

## Conflict of interest

The authors declare that the research was conducted in the absence of any commercial or financial relationships that could be construed as a potential conflict of interest.

## Publisher’s note

All claims expressed in this article are solely those of the authors and do not necessarily represent those of their affiliated organizations, or those of the publisher, the editors and the reviewers. Any product that may be evaluated in this article, or claim that may be made by its manufacturer, is not guaranteed or endorsed by the publisher.

## References

[1] Peng X. The 6pm struggle: the changing meaning of work, a culture of overtime work, and corporate governmentality in urban China. Asian Anthropol. (2020) 19:39–52. doi: 10.1080/1683478X.2019.1654499

[2] Kang JH Matusik JG Barclay LA. Affective and normative motives to work overtime in Asian organizations: four cultural orientations from Confucian ethics. J Bus Ethics. (2017) 140:115–30. doi: 10.1007/s10551-015-2683-4

[3] Liu B Chen H Yang X Hou C. Why work overtime? A systematic review on the evolutionary trend and influencing factors of work hours in China. Front Public Health. (2019) 7:343. doi: 10.3389/fpubh.2019.00343, PMID: 31803708 PMC6872522

[4] Cooke F. HRM, Work and Employment in China. London: Routledge (2005).

[5] Liu B Chen H Hou C Wang Y. The structure and measurement of overtime work: a scale development study among Chinese employees. Curr Psychol. (2021) 4:1–11. doi: 10.1007/s12144-020-01259-1

[6] Tsai MC Nitta M Kim SW Wang W. Working overtime in East Asia: convergence or divergence? J Contemp Asia. (2016) 46:700–22. doi: 10.1080/00472336.2016.1144778

[7] Gottfried AE. Intrinsic motivation in young children. Young Child. (1983) 39:64–73.

[8] Deci EL Ryan RM. Intrinsic Motivation and Self-Determination in Human Behavior. New York, NY: Plenum Publishing Co (1985).

[9] Deci EL Eghrari H Patrick BC Leone DR. Facilitating internalization: the self-determination theory perspective. J Pers. (2010) 62:119–42. doi: 10.1111/j.1467-6494.1994.tb00797.x, PMID: 8169757

[10] Zhang L. Lean production and labor controls in the Chinese automobile industry in an age of globalization. Int Labor Work Class Hist. (2008) 73:24–44. doi: 10.1017/S0147547908000033

[11] Zeng X Lu L Idris S. Working Time in Transition: The Dual Task of Standardization and Flexibilization in China. Geneva: International Labour Organization, Conditions of Work and Employment Series No. 11 (2005).

[12] Diener E. Subjective well-being. Psychol Bull. (1984) 95:542–75. doi: 10.1037/0033-2909.95.3.542 6399758

[13] Diener E Oishi S Tay L. Advances in subjective well-being research. Nat Hum Behav. (2018) 2:253–60. doi: 10.1038/s41562-018-0307-6 30936533

[14] Song YJ Lee YS. Work hours, work schedules, and subjective well-being in Korea. Int Sociol. (2021) 36:25–48. doi: 10.1177/0268580920949724

[15] Staines GL Pleck JH. Nonstandard work schedules and family life. J Appl Psychol. (1984) 69:515–23. doi: 10.1037/0021-9010.69.3.515

[16] Karhula K Wöhrmann AM Brauner C Härmä M Kivimäki M Michel A. Working time dimensions and well-being: a cross-national study of Finnish and German health care employees. Chronobiol Int. (2020) 37:1312–24. doi: 10.1080/07420528.2020.1778716 32727224

[17] Barnett RC Gareis KC Brennan RT. Fit as a mediator of the relationship between work hours and burnout. J Occup Health Psychol. (1999) 4:307–17. doi: 10.1037/1076-8998.4.4.307, PMID: 10526835

[18] Yu J Leka S. Where is the limit for overtime? Impacts of overtime on employees’ mental health and potential solutions: a qualitative study in China. Front Psychol. (2022) 13:976723. doi: 10.3389/fpsyg.2022.976723, PMID: 36600714 PMC9806229

[19] Badri M Al Khaili M Aldhaheri H Yang G Al Bahar M Al Rashdi A. Examining the structural effect of working time on well-being: evidence from Abu Dhabi. Soc Sci Humanit Open. (2022) 6:100317. doi: 10.1016/j.ssaho.2022.100317

[20] Ahn T. Reduction of working time: does it lead to a healthy lifestyle? Health Econ. (2016) 25:969–83. doi: 10.1002/hec.3198, PMID: 25974857

[21] Okamoto S. Hours of work and health in Japan. Ann Epidemiol. (2019) 33:64–71. doi: 10.1016/j.annepidem.2019.02.003 30879967

[22] Tsutsumi A. Preventing overwork-related deaths and disorders-needs of continuous and multi-faceted efforts. J Occup Health. (2019) 61:265–6. doi: 10.1002/1348-9585.12062, PMID: 31050094 PMC6620748

[23] Sparks K Cooper CL Fried Y Shirom A. The effects of hours of work on health: a meta-analytic review. J Occup Organ Psychol. (1997) 70:391–408. doi: 10.1111/j.2044-8325.1997.tb00656.x

[24] Van der Hulst M. Long workhours and health. Scand J Work Environ Health. (2003) 29:171–88. doi: 10.5271/sjweh.720 12828387

[25] Caruso C Hitchcock E Dick R Russo J Schmit J. Overtime and Extended Work Shifts: Recent Findings on Illnesses, Injuries and Health Behaviors. Cincinnati OH: NIOSH Publications Dissemination (2004).

[26] Müller G Tisch A Wöhrmann AM. The impact of long working hours on the health of German employees. Ger J Hum Resour Manag. (2018) 32:217–35. doi: 10.1177/2397002218786020

[27] Qiu D Li Y Li R He J Ouyang F Luo D. Long working hours, work-related stressors and sleep disturbances among Chinese government employees: a large population-based follow-up study. Sleep Med. (2022) 96:79–86. doi: 10.1016/j.sleep.2022.05.005, PMID: 35613538

[28] Takahashi M. Sociomedical problems of overwork-related deaths and disorders in Japan. J Occup Health. (2019) 61:269–77. doi: 10.1002/1348-9585.12016, PMID: 30977205 PMC6620752

[29] Sung H Kim J Kim J Punnett L Lee H Kim S. Association between extremely long working hours and musculoskeletal symptoms: a nationwide survey of medical residents in South Korea. J Occup Health. (2020) 62:e12125. doi: 10.1002/1348-9585.12125, PMID: 32515892 PMC7193152

[30] Ervasti J Pentti J Nyberg ST Shipley MJ Leineweber C Sørensen JK. Long working hours and risk of 50 health conditions and mortality outcomes: a multicohort study in four European countries. Lancet Reg Health Eur. (2021) 11:100212. doi: 10.1016/j.lanepe.2021.100212 34917998 PMC8642716

[31] Kivimaki M Jokela M Nyberg ST Singh-Manoux A Fransson EI Alfredsson L. Long working hours and risk of coronary heart disease and stroke: a systematic review and meta-analysis of published and unpublished data for 603,838 individuals. Lancet. (2015) 386:1739–46. doi: 10.1016/S0140-6736(15)60295-1, PMID: 26298822

[32] Inoue Y Yamamoto S Stickley A Kuwahara K Miyamoto T Nakagawa T. Overtime work and the incidence of long-term sickness absence due to mental disorders: a prospective cohort study. J Epidemiol. (2021) 32:283–9. doi: 10.2188/jea.JE20200382 33518590 PMC9086305

[33] Virtanen M Ferrie JE Singh-Manoux A Shipley MJ Stansfeld SA Marmot MG. Long working hours and symptoms of anxiety and depression: a 5-year follow-up of the Whitehall ii study. Psychol Med. (2011) 41:2485–94. doi: 10.1017/S0033291711000171, PMID: 21329557 PMC3095591

[34] Ma X. Impact of long working hours on mental health: evidence from China. Int J Environ Res Public Health. (2023) 20:1641. doi: 10.3390/ijerph20021641, PMID: 36674394 PMC9866749

[35] Virtanen M Jokela M Madsen IE Hanson LLM Lallukka T Nyberg ST. Long working hours and depressive symptoms: systematic review and meta-analysis of published studies and unpublished individual participant data. Scand J Work Environ Health. (2018) 44:239–50. doi: 10.5271/sjweh.3712, PMID: 29423526

[36] Lee K Suh C Kim JE Park JO. The impact of long working hours on psychosocial stress response among white-collar workers. Ind Health. (2017) 55:46–53. doi: 10.2486/indhealth.2015-0173, PMID: 27498571 PMC5285313

[37] Afonso P Fonseca M Pires JF. Impact of working hours on sleep and mental health. Occup Med. (2017) 67:377–82. doi: 10.1093/occmed/kqx054 28575463

[38] Brauner C Wöhrmann AM Michel A. Work availability types and well-being in Germany–a latent class analysis among a nationally representative sample. Work Stress. (2022) 36:251–73. doi: 10.1080/02678373.2021.1969475

[39] Karhula K Wöhrmann AM Brauner C Härmä M Kivimäki M Michel A. Working time dimensions and well-being: a cross-national study of Finnish and German health care employees. Chronobiol Int. (2020) 37:1312–24. doi: 10.1080/07420528.2020.1778716, PMID: 32727224

[40] Deci EL Ryan RM. The “what” and “why” of goal pursuits: human needs and the self-determination of behavior. Psychol Inq. (2000) 11:227–68. doi: 10.1207/S15327965PLI1104_01

[41] Yu S Levesque-Bristol C Maeda Y. General need for autonomy and subjective well-being: a meta-analysis of studies in the US and East Asia. J Happiness Stud. (2018) 19:1863–82. doi: 10.1007/s10902-017-9898-2

[42] Ford M Wang YR Huh Y. Work, The Work-Family Interface, and Subjective Well-Being. Handbook of Well-Being. Salt Lake City, UT: DEF Publishers (2018).

[43] Wu CH Luksyte A Parker SK. Overqualification and subjective well-being at work: the moderating role of job autonomy and culture. Soc Indic Res. (2015) 121:917–37. doi: 10.1007/s11205-014-0662-2

[44] Ghazzawi R Bender M Daouk-Öyry L van de Vijver FJ Chasiotis A. Job crafting mediates the relation between creativity, personality, job autonomy and well-being in Lebanese nurses. J Nurs Manag. (2021) 29:2163–74. doi: 10.1111/jonm.13357, PMID: 33960053 PMC8596648

[45] Bastida M Neira I Lacalle-Calderon M. Employee’s subjective-well-being and job discretion: designing gendered happy jobs. Eur Res Manag Bus Econ. (2022) 28:100189. doi: 10.1016/j.iedeen.2021.100189

[46] Neugarten BL Havighurst RJ Tobin SS. The measurement of life satisfaction. J Gerontol. (1961) 16:134–43. doi: 10.1093/geronj/16.2.134 13728508

[47] George LK. Subjective well-being: conceptual and methodological issues In: Eisdorfer C, editor. Annual Review of Gerontology and Geriatrics, vol. 2. New York: Springer (1981)

[48] Lawton MP. Environment and other determinants of well-being in older people. Gerontologist. (1983) 23:349–57. doi: 10.1093/geront/23.4.349, PMID: 6352420

[49] Diener E Suh EM Lucas RE Smith HL. Subjective well-being: three decades of progress. Psychol Bull. (1999) 125:276–302. doi: 10.1037/0033-2909.125.2.276

[50] Radloff LS. The CES-D scale: a self-report depression scale for research in the general population. Appl Psychol Meas. (1977) 1:385–401. doi: 10.1177/014662167700100306

[51] Boey KW. Cross-validation of a short form of the CES-D in Chinese elderly. Int J Geriatr Psychiatry. (1999) 14:608–17. doi: 10.1002/(SICI)1099-1166(199908)14:8<608::AID-GPS991>3.0.CO;2-Z, PMID: 10489651

[52] Yang W Xiong G Garrido LE Zhang JX Wang MC Wang C. Factor structure and criterion validity across the full scale and ten short forms of the CES-D among Chinese adolescents. Psychol Assess. (2018) 30:1186–98. doi: 10.1037/pas0000559, PMID: 29658726

[53] Allen H Slavin T Bunn W. Do long workhours impact health, safety, and productivity at a heavy manufacturer? J Occup Environ Med. (2007) 49:148–71. doi: 10.1097/JOM.0b013e31802f09ee 17293756

[54] Nie P Otterbach S Sousa-Poza A. Long work hours and health in China. China Econ Rev. (2015) 33:212–29. doi: 10.1016/j.chieco.2015.02.004

[55] Sparks K Faragher B Cooper CL. Well-being and occupational health in the 21st century workplace. J Occup Organ Psychol. (2001) 74:489–509. doi: 10.1348/096317901167497

[56] Ngai P. Global production, company codes of conduct, and labor conditions in China: a case study of two factories. China J. (2005) 54:101–13. doi: 10.2307/20066068

[57] Ferrer-I-Carbonell A. Income and well-being: an empirical analysis of the comparison income effect. J Public Econ. (2005) 89:997–1019. doi: 10.1016/j.jpubeco.2004.06.003

[58] Schneider SM. Income inequality and subjective wellbeing: trends, challenges, and research directions. J Happiness Stud. (2016) 17:1719–39. doi: 10.1007/s10902-015-9655-3

[59] Albrecht SC Kecklund G Rajaleid K Leineweber C. The longitudinal relationship between control over working hours and depressive symptoms: results from SLOSH, a population-based cohort study. J Affect Disord. (2017) 215:143–51. doi: 10.1016/j.jad.2017.03.010, PMID: 28324780

[60] Nijp HH Beckers DG Geurts SA Tucker P Kompier MA. Systematic review on the association between employee worktime control and work-non-work balance, health and well-being, and job-related outcomes. Scand J Work Environ Health. (2012) 38:299–313. doi: 10.5271/sjweh.3307, PMID: 22678492

[61] Golden L Wiens-Tuers B. Overtime work and wellbeing at home. Rev Soc Econ. (2008) 66:25–49. doi: 10.1080/00346760701668495

[62] Michelacci C. Pijoanmas J. (2007). The Effects of Labor Market Conditions on Working Time: The US-EU Experience. CEPR Discussion Paper No. DP6314. Available at: https://ssrn.com/abstract=1136621 (Accessed May, 2007).

[63] Barrick MR Mount MK. The big five personality dimensions and job performance: a meta-analysis. Pers Psychol. (1991) 44:1–26. doi: 10.1111/j.1744-6570.1991.tb00688.x

[64] Watanabe M Yamauchi K. Psychosocial factors of overtime work in relation to work-nonwork balance: a multilevel structural equation modeling analysis of nurses working in hospitals. Int J Behav Med. (2016) 23:492–500. doi: 10.1007/s12529-016-9563-x, PMID: 27102432

[65] Nathan AJ. Is Chinese culture distinctive? A review article. J Asian Stud. (1993) 52:923–36. doi: 10.2307/2059344

[66] Kim S Kwon K Wang J. Impacts of job control on overtime and stress: cases in the United States and South Korea. Int J Hum Resour Manag. (2022) 33:1352–76. doi: 10.1080/09585192.2020.1757738

[67] Yu X. Impacts of corporate code of conduct on labor standards: a case study of Reebok’s athletic footwear supplier factory in China. J Bus Ethics. (2008) 81:513–29. doi: 10.1007/s10551-007-9521-2

[68] Arnoštová M. Chinese overtime culture among white-collar workers in the first-tier cities. Acta Asiatica Varsoviensia. (2017) 2:7–28.

